# Supplementary material for: Computational fluid dynamics modelling of left valvular heart diseases during atrial fibrillation
Source: PeerJ. 2016 Jul 26;4:e2240. doi: 10.7717/peerj.2240 (PMC4974931; doi:10.7717/peerj.2240)
Supplement: Supplemental Information 1 — Maximum (ϑmax) and minimum (ϑmin) opening angles corresponding to the healthy and twelve diseased valve conditions used in the model. [file peerj-04-2240-s001.docx]

|  | | Mitral | | Aortic | |
| --- | --- | --- | --- | --- | --- |
|  |  | *ϑ_min_* [°] | *ϑ_max_* [°] | *ϑ_min_* [°] | *ϑ_max_* [°] |
| **Healthy** | | 0 | 75 | 0 | 75 |
| **AS** | Mild | 0 | 75 | 0 | 61.58 |
|  | Moderate | 0 | 75 | 0 | 54.15 |
|  | Severe | 0 | 75 | 0 | 49.58 |
| **MS** | Mild | 0 | 57.91 | 0 | 75 |
|  | Moderate | 0 | 50.99 | 0 | 75 |
|  | Severe | 0 | 46.72 | 0 | 75 |
| **AR** | Mild | 0 | 75 | 25.27 | 75 |
|  | Moderate | 0 | 75 | 33.46 | 75 |
|  | Severe | 0 | 75 | 38.08 | 75 |
| **MR** | Mild | 28.48 | 75 | 0 | 75 |
|  | Moderate | 35.07 | 75 | 0 | 75 |
|  | Severe | 38.73 | 75 | 0 | 75 |

**Table S1. Opening angles of the simulated configurations.** Maximum (*ϑ_max_*) and minimum (*ϑ_min_*) opening angles corresponding to the healthy and twelve diseased valve conditions used in the model.
