# Supplementary material for: Computational fluid dynamics modelling of left valvular heart diseases during atrial fibrillation
Source: PeerJ. 2016 Jul 26;4:e2240. doi: 10.7717/peerj.2240 (PMC4974931; doi:10.7717/peerj.2240)
Supplement: Supplemental Information 2 — Mean values of computed severity indexes of the different simulated valvulopathies during AF. Regurgitant volumes are reported as absolute values. [file peerj-04-2240-s002.docx]

|  | **Stenosis**  ***MPG* [mmHg]** | | **Regurgitation**  ***RV* [ml/beat]** | |
| --- | --- | --- | --- | --- |
|  | AS | MS | AR | MR |
| **Mild** | 25.68 | 5.74 | 27.43 | 34.00 |
| **Moderate** | 54.19 | 9.93 | 60.33 | 64.09 |
| **Severe** | 86.84 | 14.32 | 83.12 | 84.08 |

**Table S2. Severity indexes for stenosis and regurgitation.** Mean values of computed severity indexes of the different simulated valvulopathies during AF. Regurgitant volumes are reported as absolute values.
